# Toward the Reconstitution of a Two-Enzyme Cascade for Resveratrol Synthesis on Potyvirus Particles

**DOI:** 10.3389/fpls.2016.00089

**Published:** 2016-02-10

**Authors:** Jane Besong-Ndika, Matti Wahlsten, Daniela Cardinale, Jan Pille, Jocelyne Walter, Thierry Michon, Kristiina Mäkinen

**Affiliations:** ^1^Division of Microbiology and Biotechnology, Department of Food and Environmental Sciences, University of HelsinkiHelsinki, Finland; ^2^UMR 1332 Biologie du Fruit et Pathologie, INRA-Université BordeauxVillenace d’Ornon, France; ^3^Bio-Organic Chemistry, Radboud UniversityNijmegen, Netherlands

**Keywords:** enzyme immobilization, z33-peptide, antibodies, enzyme nano-carriers, virus nanoparticles, potyvirus, resveratrol

## Abstract

The highly ordered protein backbone of virus particles makes them attractive candidates for use as enzyme nano-carriers (ENCs). We have previously developed a non-covalent and versatile approach for adhesion of enzymes to virus particles. This approach makes use of z33, a peptide derived from the B-domain of *Staphylococcus aureus* protein A, which binds to the Fc domain of many immunoglobulins. We have demonstrated that with specific antibodies addressed against the viral capsid proteins (CPs) an 87% coverage of z33-tagged proteins can be achieved on potyvirus particles. 4-coumarate coenzyme A ligase (4CL2) and stilbene synthase (STS) catalyze consecutive steps in the resveratrol synthetic pathway. In this study, these enzymes were modified to carry an N-terminal z33 peptide and a C-terminal 6xHis tag to obtain ^z^4CL2^His^ and ^z^STS^His^, respectively. A protein chimera, ^z^4CL2::STS^His^, with the same modifications was also generated from the genetic fusion of both mono-enzyme encoding genes. All z33 enzymes were biologically active after expression in *Escherichia coli* as revealed by LC-MS analysis to identify resveratrol and assembled readily into macromolecular complexes with *Potato virus A* particles and α-PVA CP antibodies. To test simultaneous immobilization-purification, we applied the double antibody sandwich – ELISA protocol to capture active z33-containg mono-enzymes and protein chimera directly from clarified soluble cell lysates onto the virus particle surface. These immobilized enzymes were able to synthesize resveratrol. We present here a bottom up approach to immobilize active enzymes onto virus-based ENCs and discuss the potential to utilize this method in the purification and configuration of nano-devices.

## Introduction

The tremendous progresses made in molecular biology have opened up possibilities for building new bioinspired objects for nanotechnologies. Amongst them is the ability to reposition biocatalysts in an environment mimicking their genuine working place, the cell. For instance, metabolic pathways are often defined as a cascade of enzymatic reactions catalyzed by a sequence of neighboring enzymes. Mimicking this organization gives access to potential applications, for instance in nano-catalysis lab-on-a-chip and biosensor devices, drug delivery vectors and nano-metrology. The bottleneck in combining several different enzymes working cooperatively comes from the difficulty in controlling their relative positional assembly on the support. This control can be achieved by coupling the enzymes of interest with a compatible highly ordered protein scaffold. Within cells multi-enzyme complexes allow channeling of the substrates from one enzyme to another hence minimizing their free diffusion. This arrangement increases the efficiency of the consecutive reactions, protects the intermediates, prevents unwanted side reactions and concentrates the catalysis in one location. The influence of distance on multi-enzyme systems was demonstrated with glucose oxidase (GOx) and horse radish peroxidase (HRP) by spatially positioning them on various DNA scaffolds. The concentration of H_2_O_2_, product of the first reaction in the cascade, decreased when the distance between GOx and HRP increased, which resulted in lower activity of HRP (Fu et al., 2012). Also, functional biomimetic three-enzyme cascades have been built in polymersome nano-reactors (van Dongen et al., 2009). Scaffolding of enzymes may further improve the enzyme’s stability, activity, selectivity and specificity (Rodrigues et al., 2013). Moreover, it enables enzyme reusability (Garcia-Galan et al., 2011) whilst facilitating its simultaneous immobilization and purification (Barbosa et al., 2015). For example, a synthetic protein scaffold interacting with the enzymes in a biosynthetic pathway in a programmable manner improved production of mevalonate (Dueber et al., 2009) and glucaric acid (Moon et al., 2010) over the control. In addition, a synthetic metabolon of three enzymes, triose phosphate isomerase (TIM), aldolase (ALD) and fructose 1,6-biphosphatase (FBP), showed improved activity compared with that of the free enzymes, due to increased substrate channeling resulting from the close proximity of the enzymes (You and Zhang, 2013). This metabolon was synthesized by simultaneous immobilization and purification of the cascade enzymes from cell extracts.

Virus particles are supramolecular edifices unsurpassed in nature which are being exploited as enzyme nano-carriers (ENCs; Cardinale et al., 2012). The simplest of these virus particles constitute a combination of proteins and nucleic acids, which are precisely arranged in space. Indeed, the symmetrical arrangement of the virus particles, and the repetitive nature of their capsid protein (CP) subunits provide a chemically uniform polyvalent binding surface for immobilization of various enzymes. Furthermore, the diversity in architecture, protein composition and size ensures the availability of various structural, chemical and physical properties to select from in virus nanoparticles (VNPs) design (Besong-Ndika et al., 2015). Coupling enzymes to the highly ordered protein backbones of viruses is an attractive way to achieve positional control (Steinmetz and Evans, 2007). Many strategies have been developed to modify VNPs to allow attachment or encapsulation of proteins and other molecules (Comellas-Aragones et al., 2007; Koudelka and Manchester, 2010).

Considering enzyme patterning on solid supports, it appears that ENCs are easier to position on a support than pools of isolated enzymes. The last developments of top-down technologies enable a precise patterning of single nano-objects such as virus particles or DNA molecules on various supports. For instance, the building of pre-organized enzymatic cascades on the virus surface can be followed by top-down processes such as nanolithography or convective-capillary deposition (Cerf et al., 2011). This illustrates how bottom-up and top-down approaches begin to converge for the preparation of smart materials and bridge the gaps between the mesoscale, the microscale, and higher.

**FIGURE 1:**
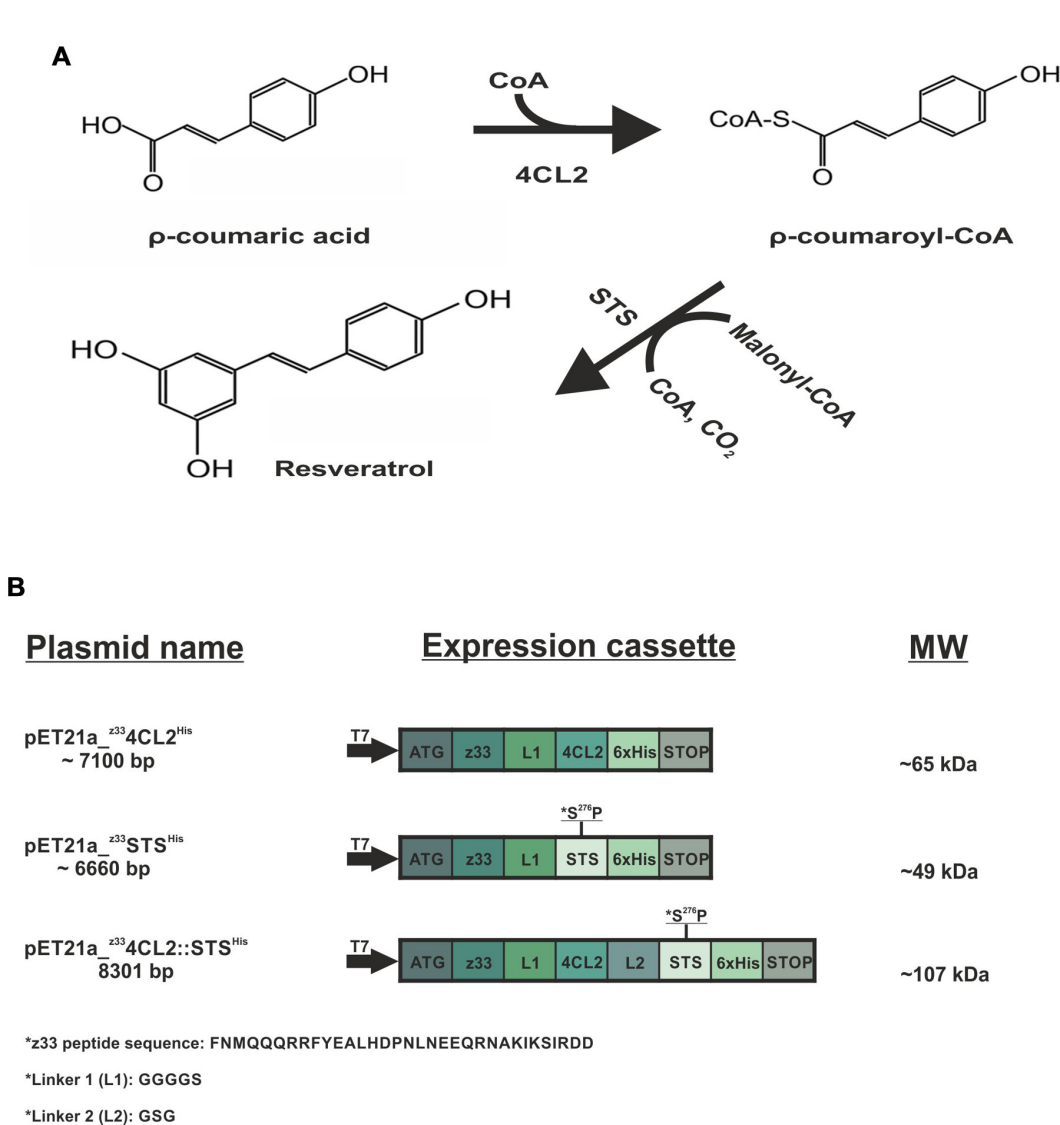
**Schematic representation of the resveratrol biosynthesis pathway and the expression cassette of the recombinant proteins. (A)** Resveratrol synthetic pathway. The ρ-coumaric acid precursor, in the presence of CoA is converted to ρ-coumaroyl-CoA by the action of 4-coumarate:coenzyme A ligase (4CL2). Subsequently, stilbene synthase (STS) in the presence of three acetyl groups from malonyl-CoA catalyzes the condensation and cyclization reaction to produce resveratrol. **(B)** Showcases the plasmids utilized in this study, the expression cassettes and the molecular weight (MW) of the recombinant proteins. The z33-tagged proteins were cloned into pET21a (+) with an N-terminal T7 promoter and a C-terminal 6x His tag. The protein sequence of z33 peptide is represented as well as the linkers used.

Four-coumarate-CoA ligase (4CL2) and stilbene synthase (STS) are enzymes involved in a cascade reaction which leads to the production of resveratrol. Resveratrol (3, 5, 4′-trihydroxy-*trans*-stilbene) is a polyphenolic compound produced by some plants in response to various infections or environmental stresses. In recent years, resveratrol has received a lot of attention due to its numerous health benefits. It is a component of grape and thought to be responsible for the cardio-protective effect of red wine (Tome-Carneiro et al., 2013). It is obtained from ρ-coumaric acid, which in the presence of co-enzyme A is converted to coumaroyl-CoA by 4CL2. Subsequently, STS adds three acetyl units from malonyl-CoA to coumaroyl-CoA followed by a cyclization reaction to produce *trans*-resveratrol (**Figure 1A**). Resveratrol production from *p*-coumaric acid has been achieved in *Escherichia coli* and *Saccharomyces cerevisiae* expressing either monomeric 4CL2 and STS (Beekwilder et al., 2006; Lim et al., 2011) or alternatively, a fusion protein resulting from a genetic fusion of these two enzymes (Zhang et al., 2006). In a previous work, we demonstrated that 4CL2 can be attached in an active form to the external surface of *Zucchini yellow mosaic virus* (ZYMV; genus *Potyvirus*) via anti-ZYMV antibodies (Pille et al., 2013). We developed an adaptable tagging strategy using a 33 - amino acid peptide (z33) derived from *Staphylococcus aureus* protein A (SpA), which binds with high affinity (*K*_d_ value 10–50 nM) to the Fc domain of immunoglobulins (Braisted and Wells, 1996). In the current study we aimed at building a 4CL2 and STS enzymatic cascade reaction on the surface of a potyviral particle. The filamentous phytovirus *Potato virus A* (PVA), which is a member of the genus *Potyvirus* was used as a model ENC. Potyviruses are plant viruses with flexible rod-shaped particles (ca. 750 nm long, 15 nm diameter) enclosing a single-stranded, polyadenylated, positive-sense genomic RNA. The virus particle is made up of about 2000 self-assembled identical coat protein subunits against which we directed the enzyme assembly. We present here a bottom up approach in which active ^z^4CL^His^ and ^z^STS^His^ or a protein chimera, ^z^4CL2::STS^His^, were captured from clarified soluble cell lysates on to the surface of PVA particles and demonstrate that resveratrol synthesis can be reconstituted with these enzymes on potyvirus particles.

## Materials and Methods

### Plasmid Constructs

The 4CL2 and STS proteins used in this study were from *Nicotiana tabacum* (GenBank accession no. U50846) and *Vitis vinifera*, respectively (GenBank accession no. EU156062). A z33 sequence (Braisted and Wells, 1996), was incorporated into the N-terminus of all proteins and cloned into a pET21a (+) –based expression vector with a C-terminal 6x His-tag. The expression clone ^z^4CL2^His^ is the same used in Pille et al. (2013).

For preparation of the ^z^STS^His^ expression clone, the pET21a (+)-z33-mYFP (Pille et al., 2013) was linearized (NEB enzymes BamHI and HindIII), gel-purified and ligated to the *STS* gene. Prior to ligation, corresponding sites were inserted into the *sts* gene via PCR using the forward primer: 5′- TCATAAGGATCC**ATGGCTTCAGTCGAGGAAATTAGA**-3′ and reverse primer: 5′- CCGTCCGAAGCTT**ATTTGTAACCATAGGAATGCTAT**-3′; BamHI and HindIII restriction sites are underlined and the corresponding *STS* sequences are shown in bold.

The ^z^4CL2::STS^His^ clone was produced via homologous recombination in yeast. A short linker of three amino acids, Glycine-Serine-Glycine, was inserted between both protein domains as in (Zhang et al., 2006). The pET21a (+)-z33-4CL plasmid was used as a template (Pille et al., 2013). The following primer pair was used to amplify *sts* from pET21a-z33-STS with insertion of a linker and the corresponding 4CL2 sequences: forward primer; CTGGCTGCTGGGCTTCCAAATGGATCTGGCatggcttcagtcgaggaaattagaaacg and reverse primer; CTCAGTGGTGGTGGTGGTGGTGATTTGTAACCATAGGAATGCTATG. The following primers were used to linearize the template plasmid, pET21a-z33-4CL, to enable insertion of the foreign DNA fragment: reverse primer; ATTTGGAAGCCCAGCAGCCAG and forward primer CACCACCACCACCACCACTG. All PCR products were cleaned up using the PCRapace kit (Invitek). Competent *S. cerevisiae* (strain YPH501) were transformed with these PCR products for homologous recombination. Colonies were selected and grown in CAU medium (synthetic-defined base medium plus tryptophan) for about 30 h. Plasmids purified from these overnight cultures were subsequently used to transform XL1-Blue cells and positive clones for downstream applications were then selected via restriction digestion and sequencing. However, the resulting plasmid was too large, about 10300 bp due to the presence of yeast replication components and hampered expression of the fusion proteins in *E. coli*. To get rid of the yeast components in the plasmid, the ^z^4CL2::STS^His^ insert was PCR-amplified with insertion of the restriction sites NheI at the N-terminus and XhoI at the C-terminus and cloned into the pET21a (+) vector. The primers used to amplify the insert were: forward primer ACATATGGCTAGCTTCAACATGCAGCAGC and reverse primer GGTGGTGCTCGAGATTTGTAACCATAGG. After sequencing, positive clones were used to transform BL21 (DE3) cells.

All the proteins contained a linker, GGGGS, at the C-terminal of z33 peptide to ensure flexibility (Pille et al., 2013). The expression cassettes of all proteins are represented in **Figure 1B**.

### Clarified Cell Lysate Preparation

*Escherichia coli* BL21 (DE3) cells were transformed with expression vectors harboring the z33-containing proteins. Expression was performed in 1 l 2x LB medium supplemented with 100 mg/ml ampicillin. Bacteria cultures were grown until OD_600_ 1.0 followed by induction with 1 mM isopropyl β-D-thiogalactoside (IPTG) for about 18 h at 20°C. Cells were harvested by centrifugation at 6000 rpm for 10 min at 4°C. Pellets were re-suspended in lysis buffer (25 mM NaH_2_PO_4_, 100 mM NaCl, 5% glycerol pH 8.0) containing 1 mM PMSF, 1 mg/ml lysozyme and 1 protease inhibitor mini tablet (ThermoScientific) followed by 1 h incubation at 4°C. For ^z^STS^His^, the lysis buffer was supplemented with 20 mM β-mercaptoethanol to reduce oxidation damage. Cells were lysed by sonication for a total of 10 min (30 s burst, 30 s cooling, 40% power cycle, power level 2, 0.7 duty cycle) using the Labsonic U sonicator (BRAUM). Cell debris was removed by centrifugation at 14000 *g* for 30 min at 4°C. Protein expression was confirmed by western blot analysis. Aliquots of the clarified cell lysates were stored at -20°C. Untransformed empty BL21 lysate was also prepared as above for use as a negative control.

### Protein Purification

The purification of ^z^4CL2^His^ and ^z^STS^His^ was performed under native conditions as previously described for ^z^4CL2^His^ (Pille et al., 2013). The fusion protein ^z^4CL2::STS^His^ was expressed as above and purified under denaturing conditions according to the supplier’s instructions (Machery-Nagel, Protino^®^ Ni-NTA). Protein purification was performed by immobilized metal affinity chromatography (IMAC) using Ni-NTA (Ni^2+^ immobilized on nitrilotriacetic acid). The clarified lysate (about 50 ml) was allowed to bind 1 ml Ni-NTA beads overnight at 4°C after which the beads were allowed to settle in an empty column. The beads were washed four times with wash buffer (50 mM NaH_2_PO_4_, 300 mM NaCl, 20 mM imidazole, 8 M urea, pH 8.0). Proteins were then eluted with elution buffer (50 mM NaH_2_PO_4_, 300 mM NaCl, 250 mM imidazole, 8 M urea, pH 8.0) and analyzed on SDS-PAGE. To remove imidazole and urea whilst refold the proteins, the eluted proteins were extensively dialyzed against phosphate buffer (25 mM NaH_2_PO_4_, 100 mM NaCl, pH 8.0). Most of the protein precipitated during dialysis and the precipitate was removed by centrifugation at maximum speed. The remnant of protein contained in the soluble fraction was further purified by size exclusion chromatography on Sephacryl S-200 on an X16 column using the ÄKTA Prime system. Phosphate buffer was used as the eluent at a flow rate of 0.5 ml/min and 1.5 ml fractions were collected and analyzed by SDS-PAGE. All proteins were aliquoted and stored in phosphate buffer at -20°C.

### PVA Particle Purification

*Nicotiana benthamiana* plants were infected with PVA virus by mechanical inoculation or *Agrobacterium* mediated infiltration. Plants were grown under greenhouse conditions for about 3 weeks. Infected leaves were collected 1 day before and stored at 4°C. Leaves were homogenized in 2x volume of 0.1 M phosphate buffer pH 8 containing 0.15% 2-mercaptoethanol and 0.01 M EDTA (1 g of infected leaf material per 2 ml of buffer). Clarified lysate was obtained by low speed centrifugation (LSC) at 10 000 rpm for 20 min. Supernatant was filtered and triton X-100 was added to a final concentration of 3%. The mixture was stirred for 3 h at 4°C. Insoluble material was removed by LSC at 10 000 rpm for 10 min. PEG 6000 (40 g/liter of supernatant) and NaCl to a final concentration of 0.2 M were added to the supernatant and stirred for 1.5 h at 4°C. Virus particles were pelleted by LSC at 10 000 rpm for 20 min then pellets were re-suspended in 0.1 M phosphate buffer pH 8 containing 1% Triton X-100 (buffer volume should be 1/10th of the original volume of the supernatant). Virus particles were pelleted by high speed centrifugation (HSC) at 40 000 rpm at 4°C for 1 h (Beckman Ultracentrifuge). Pellets were re-suspended in 0.2 M phosphate buffer pH 8.0. Particles were further purified on 30% sucrose in 0.1 M phosphate buffer pH 8 by HSC at 90 000 g for 3 h at 4°C. Pellets were re-suspended in 2 ml of 0.1 M phosphate buffer pH 8 and again purified through a 5–40% sucrose gradient in 0.1 M phosphate buffer pH 8 by HSC at 80 000 *g* for 1 h at 4°C. Virus particles were analyzed on SDS-PAGE and protein concentration was measured using the Nanodrop^TM^ (ThermoScientific). Virus particles were stored long term at -80°C and short term at -20°C.

### α-PVA CP Antibody Purification

Recombinant PVA CP protein was analyzed by SDS-PAGE and transferred to a nitrocellulose membrane. PVA CP containing band was located by brief staining with Ponceau S and this area was excised. The protein containing strip was de-stained with 1x PBS buffer then blocked for 1 h with the same buffer containing 10% BSA at RT. Rabbit antisera against native PVA particles was diluted about 1:4 times in 1x PBS and incubated with the strip overnight at 4°C. The strip was washed three times with 1x PBS then once with ddH_2_O. Antibody was eluted from the strip four times with 400 μl 5 mM glycine-HCl pH 2.3, containing 400 mM NaCl, and immediately neutralized with 20 μl Na_2_HPO_4_. Antibody concentration was measured with a Nanodrop^TM^. Eluted fractions were pooled and dialyzed extensively against 1x PBS at 4°C. Antibody was stored at 4°C until further use.

### α-PVA : ^z^4CL2::STS^His^ Affinity Assay

Affinity assay was performed as described earlier (Pille et al., 2013) with minor modifications. IgGs and ^z^4CL2::STS^His^ fusion protein were mixed in molar ratios of 1:1, 1:3, and 1:5. Binding was allowed to proceed for 45 min at RT after which the resulting complex was purified via affinity chromatography using Ni-NTA beads as described above. IgGs treated as above were used as a negative control. Samples were analyzed by SDS-PAGE followed by silver staining.

### Macromolecular Assembly in Solution

Assembly was performed as previously described (Pille et al., 2013) with slight modifications. PVA particles were mixed with α-PVA and ^z^4CL2::STS^His^ purified under denaturing conditions (PVA CP/ α-PVA /z33-enzyme 1:1:8 ratio). All components were left to bind for 2 h at 4°C in 0.1 M sodium phosphate buffer pH 8. To eliminate any unbound components, the assembled complex was dialyzed extensively using dialysis buttons and a 300 kDa MWCO (Molecular Weight Cut Off) membrane (Spectra – Pro Biotech) for 4 days with regular buffer changes. The resulting complex and controls were resolved by SDS-PAGE and visualized by silver staining.

### DAS ELISA-Based ENC Formation

Enzyme nano-carriers were immobilized on 2 ml polypropylene tubes following the DAS (Double Antibody Sandwich) ELISA procedure. First the tubes were coated with 3.6 μg/ml of α-PVA diluted in ELISA coating buffer (Na_2_CO_3_, NaHCO_3_ pH 9.6) by incubation for 3 h at 37°C and washed three times, 3 min each with wash buffer (1x PBS containing 0.05 % Tween-20). Empty spots were blocked with 5% BSA in 1x PBS for 1 h at RT. 8 μg/ml PVA particles, diluted in sample buffer (1x PBS containing 0.1% BSA and 0.05% Tween-20) were added to the tubes and incubated overnight at 4°C. To prepare the enzyme-IgG conjugates, the clarified soluble cell lysate (protein 100 mg/ml) was incubated with 9 μg/ml α-PVA for 1 h at RT. The tubes were washed as above and their inner surface incubated with the cell lysate/α-PVA mixed overnight at 4°C. Finally, the tubes were washed extensively for about 30 min with regular buffer changes. Tubes were stored at 4°C.

Two controls were prepared in addition to this experiment. The first control was prepared exactly as above with untransformed clarified soluble cell lysates instead protein containing cell lysates. The second control contained only the initial antibody layer, the PVA particle layer and the enzyme layer instead of enzyme-anitbody layer.

### Enzyme Assay

Enzymatic reactions were performed in parallel with the same enzyme batch either immobilized or free in solution in the activity buffer containing 25 mM Na_2_HPO_4_ and 100 mM NaCl, pH 8.0. Clarified *E. coli* lysates obtained after expression of ^z^4CL2^His^ and ^z^STS^His^ were mixed in a 1:1 (100 mg/ml each) ratio. The reaction mixture contained 1 mM co-enzyme A (CoA), 0.5 mM ρ-coumaric acid, 5 mM ATP, 10 mM MgCl_2_ and 2 mM DTT and 0.5 mM malonyl-CoA in 200 μl activity buffer. The reaction was initiated by adding ρ-coumaric acid and allowed to proceed for 1 h at 28°C. The product (resveratrol) was extracted 2–3 times with 600 μl ethyl acetate (EtOAc), the organic phase was collected and the solvent was removed by centrifugal evaporation. The dried extract was re-suspended in 50% MeOH in MQ/5% formic acid and analyzed by LC-MS.

### LC-MS Analysis

Samples were injected into an Acquity UPLC system (Waters, Manchester, UK), equipped with a Cortecs C18 column (50 mm × 2.1 mm inner diameter, particle size 1.6 μm). The UPLC was operated with a flow-rate of 0.3 ml/min in gradient mode, at a temperature of 30°C. Solvents used in the gradient were A: 0.1% formic acid in water and B: 0.1% formic acid in acetonitrile. The initial conditions of the linear gradient were A: 5% and B: 95% and the conditions were changed to A: 95% and B: 5% in 5 min. Injection volumes varied from 0.1 to 5 μL. Mass spectra were recorded with a Waters Synapt G2-Si mass spectrometer (Waters, Manchester, UK). Measurements were performed using negative electrospray ionization (ESI) in resolution mode. Ions were scanned in the range from 50 to 1200 m/z. MS and MS/MS analyses were performed with scan times of 0.2 s. Capillary voltage was 2.0 kV, source temperature 120°C, sampling cone 40.0, source offset 60.0, desolvation temperature 600°C, desolvation gas flow 1000 l/h and nebulizer gas flow 6.5 Bar. Leucine-encephalin was used as a lock mass and calibration was done with sodium formiate.

### TEM Analysis of Coated PVA Particle

^z^4CL2::STS^His^ (purified under denaturing conditions) was utilized as a model enzyme to showcase the effectiveness of this strategy. Carbon coated grids were incubated with 20 μl PVA (0,0135 mg/ml) diluted in PBS-T BSA for 5 min at RT then for 1 h in 5% BSA in PBS. The grids were further incubated in a 20 μl mixture of a 1:1 ratio of 1:300 diluted α-PVA and protein for an hour. They were washed once with 20 μl BSA PBS-T (1x PBS containing 0.1% BSA and 0.05% Tween-20) for 5 min at RT. The grids were incubated in a 1:20 dilution of GAM 10 (10 nm gold labeled secondary antibody) for 1 h, washed again then stained with 3% uranyl acetate for 30 s. Visualization was done with the JEOL 1400 Electron Microscope.

## Results

### Engineering and Expression of z33-Tagged Enzymes

**FIGURE 2:**
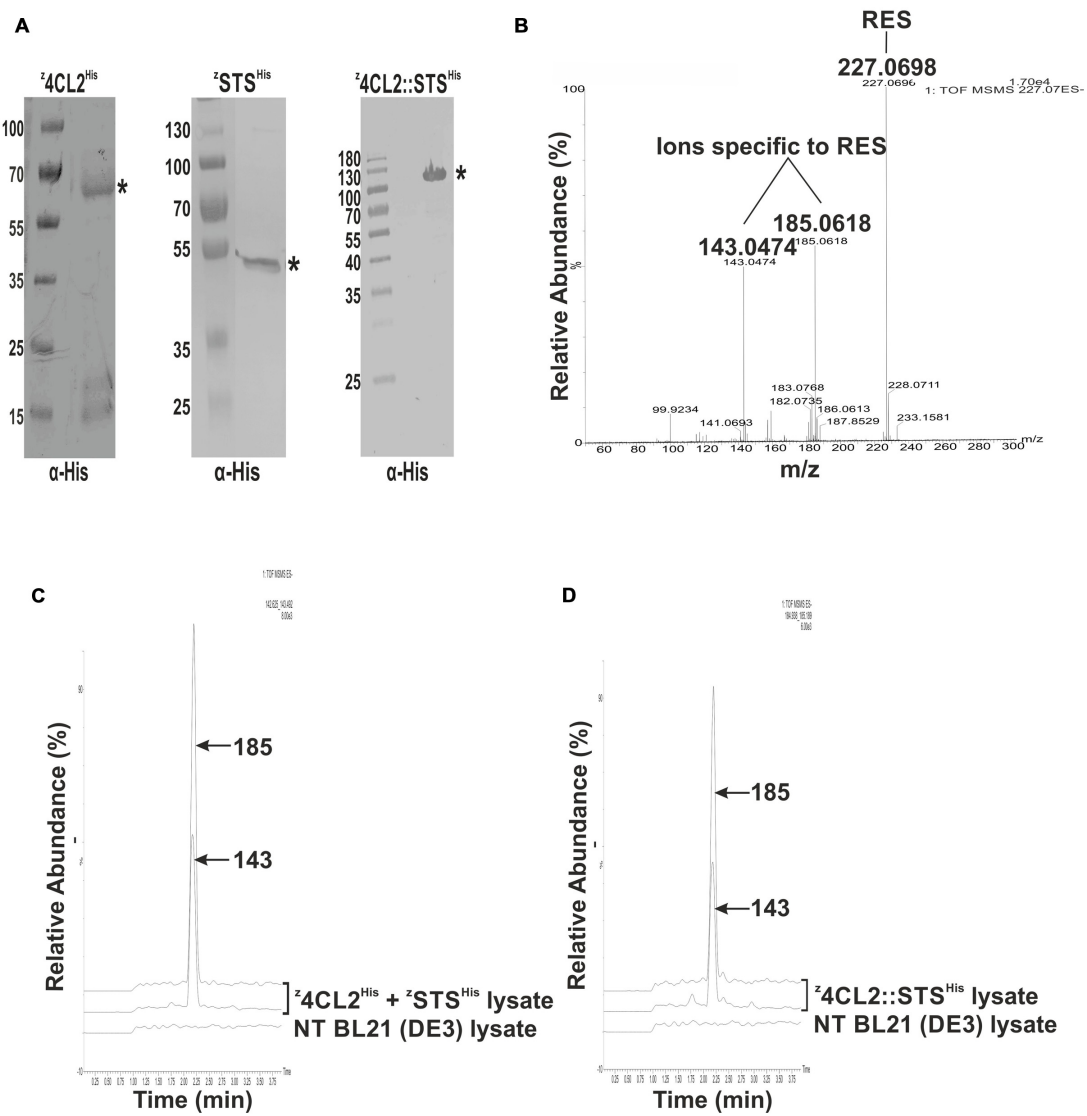
**Assessment of the activity of the expressed proteins in solution. (A)** Western blot analysis of crude cell extracts from expressed z33-tagged proteins probed with α-His. ^∗^denotes the expected MW of the expressed recombinant protein. **(B)** QTOF MS/MS spectra of a resveratrol standard showing its precursor ion at m/z 227.0698 and the daughter ions at m/z 143.0474 and m/z 185.0618 derived from its fragmentation. **(C)** Enzymatic activity from a mixture of clarified soluble cell lysates harboring ^z^4CL2^His^ and ^z^STS^His^. Resveratrol synthesis was initiated by the addition of ρ-coumaric acid to the cell lysates. Resveratrol was extracted with ethyl acetate and QTOF MSMS analysis was performed. Two peaks m/z 143 and 185 corresponding to resveratrol fragmentation ions, were eluted around 2.25 min. A non-transformed (NT) BL21(DE3) clarified cell lysate treated as above, did not contain resveratrol. **(D)** Enzymatic activity from clarified soluble cell lysate harboring the ^z^4CL2::STS^His^ protein chimera. QTOF MSMS analysis also revealed the presence of two ions (m/z 143 and 185) around 2.25 min corresponding to resveratrol daughter ions.

The enzymes used in this study, 4CL2 and STS, are involved in the resveratrol synthetic pathway (**Figure 1A**). These enzymes have successfully been expressed as soluble forms in *E. coli* (Wang et al., 2008, 2011). In this study, the z33 peptide was fused to the N-terminus of the expressed proteins and a 6x His-tag to the C-terminus (**Figure 1B**). The resulting clones, labeled ^z^4CL2^His^ and ^z^STS^His^ were expressed in BL21 (DE3) cells (**Figure 2A**). As observed also previously (Pille et al., 2013), the presence of the z33 peptide did not affect the expression of ^z^4CL2^His^. During the cloning process, an unintentional mutation was introduced into the *sts* gene leading to the S^276^P substitution. This mutation was ignored as it was not a critical amino acid residue in the active site of this chalcone synthase (CHL) -like enzyme (Jez and Noel, 2000; Suh et al., 2000). This did not rule out a possible effect of the mutation on the stability of the protein.

**FIGURE 3:**
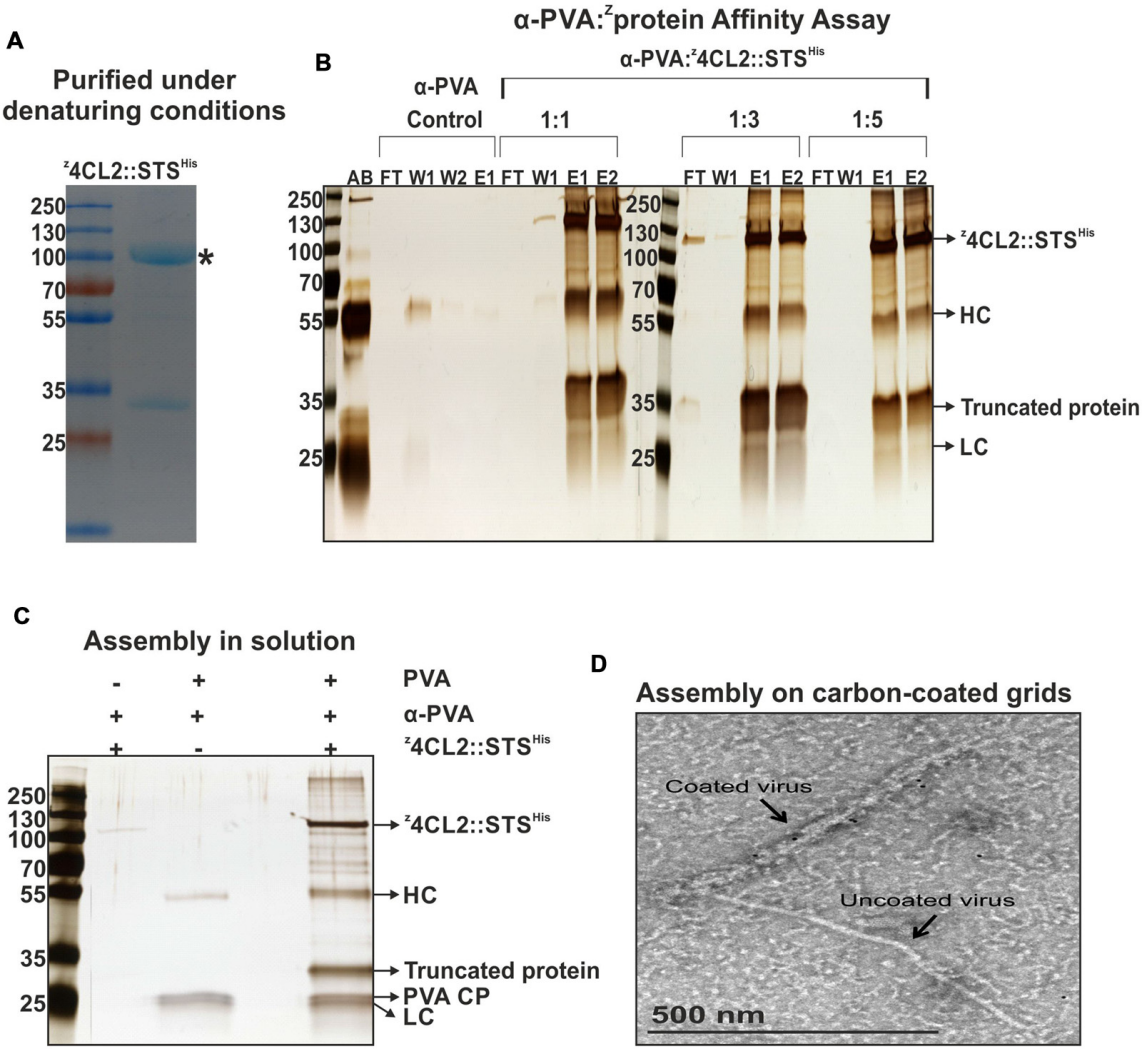
**Affinity assay and macromolecular assembly in solution and on carbon coated grids. (A)** Coomassie stained SDS-PAGE gel of purified ^z^4CL2::STS^His^. Affinity chromatography was performed using the Ni-NTA matrix (IMAC) under denaturing conditions followed by size exclusion chromatography on Sephacryl S-200. An N-terminal truncated form of ^z^4CL2::STS^His^ of 32 kDa was also identified. ^∗^denotes the expected MW of the expressed recombinant protein. **(B)** ^z^4CL2::STS^His^ and IgG affinity assay analyzed on silver stained SDS-PAGE gel. ^z^4CL2::STS^His^ was incubated with rabbit-born polyclonal PVA antibody at three different antibody to protein ratios (1:1; 1:3, and 1:5). The resulting complex was purified by IMAC. As a negative control, PVA antibodies alone were also purified via IMAC. AB, PVA antibody; FT, Flow through; W, Wash; E, Elution; HC, IgGs Heavy chain; LC, IgGs Light chains. **(C)** Silver stained SDS-PAGE gel of the macromolecular complex formed between ^z^4CL2::STS^His^, PVA antibody and PVA particles in solution. All components of the complex were incubated for an hour at RT followed by extensive dialysis for 4 days against a 300 MWCO membrane (cut off 300 kDa). Two similarly treated controls were included: the first did not contain PVA particles (Lane 2) and the second did not contain the z33-tagged protein (Lane 3). **(D)** Transmission electron microscopy depiction of PVA particles coated with ^z^4CL2::STS^His^ via PVA antibody. After particle deposition, the grid was first incubated with α-His antibodies, washed and then incubated with a 10 nm gold-conjugated antibody to probe the presence of the fusion enzyme on the virus particles. An uncoated particle can also be seen on the image.

4CL2 and STS have previously been fused genetically, interspaced by a three amino acid linker (glycine-serine-glycine) and equipped with an N-terminal 6x His-tag (Zhang et al., 2006; Wang et al., 2011). In this study, 4CL2 and STS were fused by homologous recombination in yeast and this fusion protein was tagged at its N-terminus with the z33-peptide and at its C-terminus with a 6x His-tag, to build the ^z^4CL::STS^His^ protein chimera. This protein chimera was present in the *E. coli* crude extracts after expression (**Figure 2A**). However, further analysis of the soluble and insoluble fractions revealed most of the protein was retained in inclusion bodies. The ^z^4CL2::STS^His^ protein chimera was extracted from these inclusion bodies under denaturing conditions (**Figure 3A**). Unfortunately, all dialysis driven attempts to refold this protein were unsuccessful. Also, further purification by size exclusion chromatography did not overcome the refolding hurdles as no significant enzyme activity could be detected.

### Activity of z33-Tagged Enzymes from *E. coli* Lysate

The production of resveratrol was monitored by tandem mass spectrometry (MS/MS) as previously described (Lo et al., 2007; Menet et al., 2014). Clarified soluble cell lysates containing ^z^4CL2^His^ and ^z^STS^His^ were mixed in a 1:1 (100 mg/ml each) ratio. An enzymatic assay was performed with this lysate mix and the product from the reaction was analyzed and compared to a resveratrol (RES) standard. The standard mass spectrum displayed a single product at m/z 227.07 [M-H]^-^ corresponding to the resveratrol standard and subsequent ionization in an ESI source (fragmentation) identified two daughter ions of m/z 143.0474 and 185.0618 specific to resveratrol (**Figure 2B**). The amount of resveratrol synthesized from the lysates was quite low hence a single product band could not be detected after MS analysis. However, after MS/MS analysis, two daughter ions, 143 and 185, identical to those obtained with the standard could be detected at 2.25 min (**Figure 2C**). These results demonstrated that ^z^4CL2^His^ and ^z^STS^His^ enzymes were both active in the clarified soluble lysate mix.

As above, RES synthesis was also assessed in clarified soluble *E. coli* lysate containing the ^z^4CL2::STS^His^ fusion protein. After the enzymatic assay on the lysates, tandem mass spectrometry allowed the identification of two daughter ions at m/z 143 and 185 at about 2.25 min from the product identical to the standard (**Figure 2D**). This result confirmed that both enzymes were active in the fusion protein. No RES was produced in a clarified *E. coli* lysate obtained from untransformed BL21 (DE3) cells (negative control). This affirmed the presence of RES in the samples was due to the presence of the recombinant enzymes in the cell lysate.

### The z33-Enzyme Fusion Binds to IgGs

The ^z^4CL2::STS^His^ protein chimera purified under denaturing conditions (**Figure 3A**) was used as a model protein to investigate the binding of z33 to rabbit IgGs directed toward PVA CP (α-PVA). The ^z^4CL2::STS^His^ fusion protein was mixed with rabbit IgGs in different ratios and the resulting complex was purified via affinity chromatography using Ni-NTA beads. When the antibody to protein ratio was 1:1 or 1:3, most of the antibody was bound to the fusion protein but a small amount of the constituents were detected in the flow through and/or wash fractions (**Figure 3B**). However, when the antibody to protein ratio was 1:5, all the ^z^4CL2::STS^His^ protein and IgGs were retained in the column through the Ni-NTA::His interaction.

### Decoration of PVA Particles with ^z^4CL2::STS^His^ in Solution

The next step was to investigate the binding of ^z^4CL2::STS^His^ to PVA particle surface using antibodies directed against the CP of native PVA particles (α-PVA). PVA particles were incubated with α-PVA and z33-tagged fusion protein in a molar ratio of 1:1:8 corresponding to 1 CP: 1 IgG: 8 fusion proteins. The mixture was extensively dialyzed against a 300 kDaMWCO membrane to exclude any unbound molecules. All three components assembled into macromolecular complex hence were retained in the dialysis button (**Figure 3C**, lane 4). On the other hand, in the absence of PVA particles, only a minute amount of ^z^4CL2::STS^His^ protein was retained in the buttons (**Figure 3C**, lane 2). As expected, in the absence of ^z^4CL2::STS^His^, PVA particles and α-PVA were retained in the dialysis buttons (**Figure 3C**, lane 3).

We further confirmed the coating of PVA particles with ^z^4CL2::STS^His^ by TEM imaging. An immune-conjugate composed of α-His antibody coupled to a complementary 10 nm gold bead-labeled IgG was used to demonstrate the presence of ^z^4CL2::STS^His^ on the surface of the particles. When compared to an uncoated particle (right next to the coated particle in the image), it was clear that the decorated particle displayed an additional layer of material all along its length, resulting in an increase of its width by at least a factor of 2 (**Figure 3D**). This extra layer was due to ^z^4CL2::STS^His^ - α-PVA coupling to the particles.

### Resveratrol Synthesis from Enzyme Containing PVA ENCs

In spite of the successful macromolecular assembly obtained with the ^z^4CL2::STS^His^ protein chimera purified under denaturing conditions and carried out in solution, the protein remained inactive and RES was not detected with these decorated ENCs, a plausible reason being the inability to refold the protein after denaturing purification. Consequently, we attempted to capture recombinant active enzymes directly from clarified soluble cell lysates on to PVA particles adsorbed on polypropylene tubes.

**FIGURE 4:**
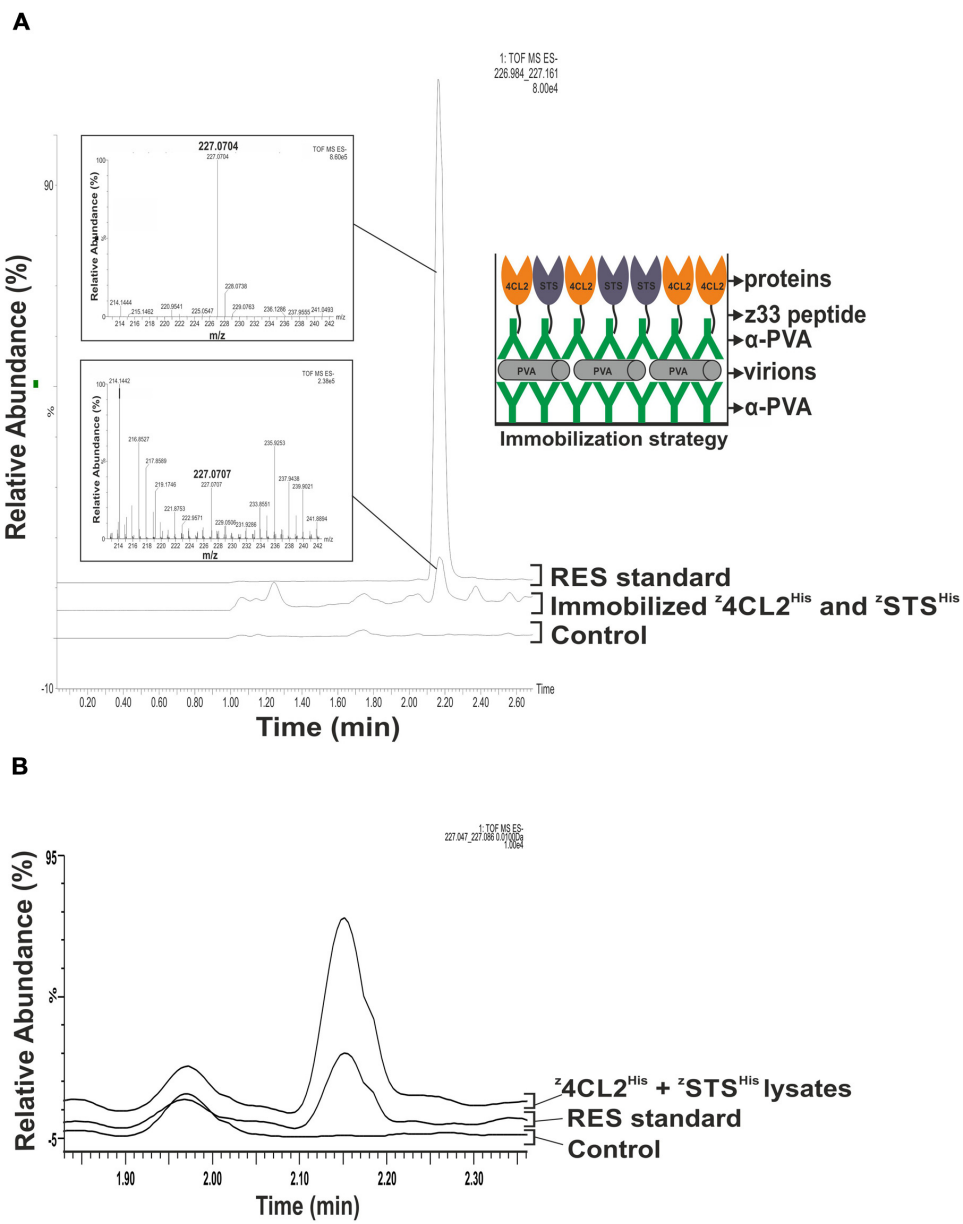
**Resveratrol synthesis from immobilized ^z^4CL2^His^ and ^z^STS^His^. (A)** Enzymatic activity from PVA immobilized ^z^4CL2^His^ and ^z^STS^His^ proteins. PVA particles were trapped in a 2 ml polypropylene tube pre-coated with PVA antibody and uncoated areas were blocked with BSA. Clarified *Escherichia coli* BL21 (DE3) lysate harboring ^z^4CL2^His^ and ^z^STS^His^, respectively, were mixed in a 1:1 ratio and incubated with PVA antibody. The antibody: protein complex was allowed to bind the trapped PVA particles overnight. Unbound components were washed out after each step. Resveratrol synthesis was initiated from the immobilized enzymes by addition of the necessary substrates followed by resveratrol extraction with ethyl acetate. QTOF MS analysis was performed to identify resveratrol. Resveratrol standard with an m/z of 227.0704 (upper inset) and eluted around 2.20 min was used as a positive control to confirm its presence in the experimental sample (lower inset). The figure on the right is a schematic representation of the macromolecular assembly. **(B)** Control experiment to investigate direct enzyme binding to initial PVA particle and antibody layer and polypropylene tubes. PVA particles were trapped in a 2 ml polypropylene tube pre-coated with PVA antibody. Clarified *E. coli* BL21 (DE3) lysate harboring ^z^4CL2^His^ and ^z^STS^His^, respectively, were mixed in a 1:1 ratio and added to the pre-coated tubes. Tubes were washed and resveratrol synthesis reactions were initiated. Resveratrol standard with an m/z of 227.0704 (upper inset) and eluted around 2.20 min obtained from an authentic standard and from clarified lysates containing ^z^4CL2^His^ and ^z^STS^His^, were used as a positive control. No resveratrol could be detected from this control.

**FIGURE 5:**
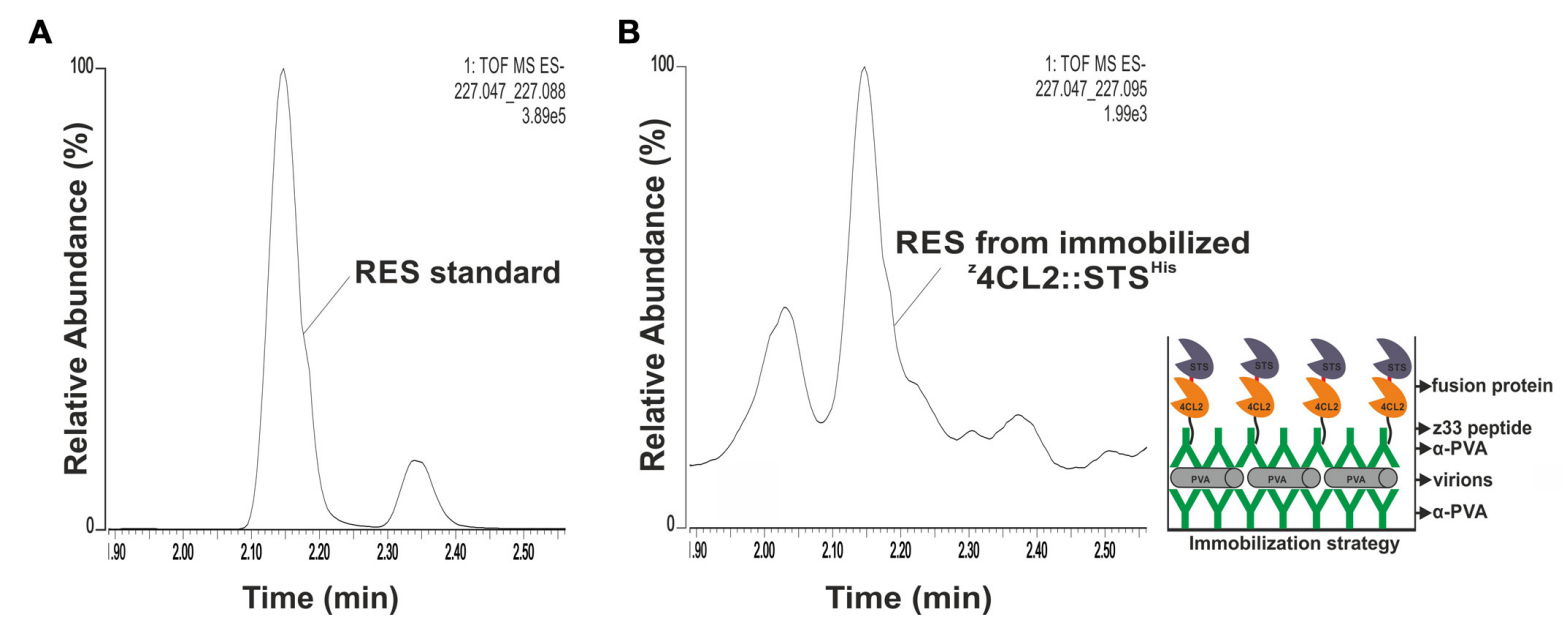
**Resveratrol synthesis from immobilized ^z^4CL2::STS^His^. (A)** QTOF MS analysis of resveratrol standard with a peak between 2.10 and 2.20 min corresponding to *trans*-resveratrol. The smaller peak might be *cis*-resveratrol. **(B)** Enzymatic activity from PVA immobilized ^z^4CL2::STS^His^ protein chimera. PVA particles were trapped on a plastic plate pre-coated with PVA antibody. Clarified *E. coli* BL21(DE3) cell lysate harboring ^z^4CL2::STS^His^ was incubated with PVA antibody and the protein: antibody complex was allowed to bind the trapped particles overnight. Unbound components were washed away after each step. Resveratrol synthesis was initiated from the immobilized enzymes by addition of the substrates and the product was extracted with ethyl acetate followed by QTOF MS analysis. The figure on the right hand side shows a schematic representation of the macromolecular assembly.

Clarified soluble cell lysates containing ^z^4CL2^His^ and ^z^STS^His^ or ^z^4CL2::STS^His^ were, respectively, incubated with α-PVA. The α-PVA and cell lysate mixes containing the mono-enzymes were added to polypropylene tubes containing immobilized PVA particles to obtain decorated ENCs (**Figure 4A**). Unbound components were removed by washing and resveratrol catalytic cascade reactions were initiated from these immobilized enzymes. LC-MS analysis of the product extract revealed a compound identical to the *trans*-resveratrol standard (m/z 227.070; **Figure 4A**, left panel). To confirm that the observed activity was due to the presence of ^z^4CL2^His^ and ^z^STS^His^, the same experiment was performed with untransformed BL21 (DE3) cells. No resveratrol was synthesized in this control sample. Furthermore, to confirm that the observed activity was from the enzymes attached on the PVA particles via the second α-PVA layer and not on plastic, the first antibody layer, or directly to PVA particles the same experiment was carried out without adding α-PVA to the clarified cell lysates containing ^z^4CL2^His^ and ^z^STS^His^. No detectable RES peak was observed from this control assembly after LC-MS analysis (**Figure 4B**) when compared to the RES standard as well as RES peak derived from the same cell lysate batch. This control confirmed no direct binding of the enzymes either to polypropylene tubes or to the first antibody layer took place after blocking them with the immobilized PVA particles. Also, it excluded the possibility of unspecific binding of the z33-tagged enzymes directly to PVA particles. Our conclusion therefore is that the detected enzyme activity was derived from the enzymes organized on the virus particle surface. A second peak with a retention time around 2 min could be seen in the controls and the samples. The content of this peak was not verified and is unknown. As with the monomeric enzymes, a peak with the same retention time and molecular mass as the RES standard peak (**Figure 5A**) was also produced in assays conducted with the immobilized ^z^4CL2::STS^His^ protein chimera (**Figure 5B**) confirming the fusion protein could be immobilized directly from the cell lysate onto PVA particles via the α-PVA antibodies in an active form.

## Discussion

In this work, we designed PVA-based ENCs displaying active ^z^4CL2^His^ and ^z^STS^His^ or a protein chimera ^z^4CL2::STS^His^ involved in RES biosynthesis.

The z33 peptide, fused to the N-terminus of all proteins, enabled antibody-mediated functionalization of PVA particles and the GGGGS peptide linker inserted into the C-terminal of the z33 peptide ensured its free movement. An addition linker, GSG, was inserted between the two protein domains in the protein chimera to avoid steric interference and 6x His-tag was also engineered to the C-terminal of the proteins of interest to enable purification. Most of the z33-tagged mono-enzymes were expressed as active, soluble proteins in *E. coli* (**Figure 2**). Nonetheless, majority of the ^z^4CL2::STS^His^ protein chimera accumulated in inclusion bodies and all attempts to purify under native conditions failed. *Arabidopsis thaliana* 4CL1, grape STS and a fusion protein 4CL::STS have previously been purified in a native and active form via an N-terminal His-tag (Wang et al., 2011). We reckoned addition of the z33 peptide to the N-terminus or the C-terminal location of the His-tag most likely caused the accumulation of the protein chimera in inclusion bodies.

The protein chimera, ^z^4CL2::STS^His^, was purified from inclusion bodies (**Figure 3A**) and although refolding was unsuccessful, this protein showed high affinity for α-PVA IgGs when the protein was supplied in fivefold excess (**Figure 3**) binding both heavy chains of the antibody. This was consistent with our earlier observation using a z33 tagged yellow fluorescent protein (Pille et al., 2013). Even though the protein was inactive, the observed binding to α-PVA IgGs indicated the z33 peptide was fully functional after the denaturing purification and still permitted the antibody-mediated absorption of this inactive protein chimera to PVA particles in solution and on carbon coated grids. Furthermore, the size of the protein, about 107 kDa, did not affect the antibody-binding property of the z33 peptide showing the robustness of this virus decoration strategy.

*Potato virus A* forms flexible rod-shaped particles composed of about 2000 coat protein subunits surrounding a single-stranded positive sense RNA molecule. Theoretically all CP subunits can be recognized by the α-PVA IgGs and consequently the z33-tagged enzymes. Transmission electron microscopy revealed only very few gold labels on the surface of the particles when detection of the antibodies bound to ^z^4CL2::STS^His^ was carried out with a secondary antibody conjugated with gold beads (**Figure 3D**). This was not surprising as we had earlier shown that the amount of beads does not correlate with the actual particle coverage and discussed a possible cause to be steric hindrance from several antibody layers and extensive washes (Pille et al., 2013). However, the EM images revealed virus particles with increased width suggesting a good coverage of PVA by ^z^4CL2::STS^His^.

Multi-enzyme systems allow channeling of the substrates from one enzyme to another, hence increasing their catalytic efficiency and immobilization of the enzymes further improves product yield. Using the double antibody sandwich enzyme-linked immunosorbent assay (DAS-ELISA) method, we built a macromolecular assembly in which PVA particles immobilized in a polypropylene tube were functionalized with active ^z^4CL2^His^ and ^z^STS^His^ (**Figure 4A**) or ^z^4CL2::STS^His^ (**Figure 5**). This method has been used previously to capture virus particles from plant sap onto polypropylene tubes pre-coated with coat protein antibodies for subsequent detection of viral RNA by RT-PCR (Fedorkin et al., 2000). More so, a similar immune-capture procedure was used prior to real-time quantitative RT-PCR to detect tobacco mosaic virus (TMV) from soil samples (Yang et al., 2012). A low amount of enzyme activity was associated with these functionalized viral ENCs. We previously showed that 4CL2 remains fully active upon fusion to z33 (Pille et al., 2013) and it has been shown that STS activity may vary tremendously depending on its source or the expression construct used (Lim et al., 2011). Based on this, we believe the second reaction in the cascade catalyzed by STS might be the rate limiting step and might offer a plausible explanation for the observed low efficiency of resveratrol synthesis. However, we show that the observed low activity was specific to enzymes attached to the virus surface and not to the initial antibody layer or the polypropylene tube (**Figure 4B**). The absence of activity when the enzymes were incubated in tubes containing only the initial antibody layer and the PVA particles indicated the PVA particle layer in addition to BSA efficiently blocked the binding surface of the initial antibody layer. It also indicated there was no unspecific interaction between the z33-tagged enzymes and the virus particle.

A 15-fold increase in RES production was obtained when a translational fusion of 4CL and STS was used as a catalyst compared to a mixture of the mono-enzyme. These activities were monitored with the enzymes free in solution (Zhang et al., 2006). The stimulation of the catalytic efficiency was attributed to the physical localization of the two active sites, interspaced by 70 Å (Wang et al., 2011). The low catalytic efficiency of our system made it near to impossible to compare the enzyme activity from ^z^4CL2^His^ and ^z^STS^His^ functionalized ENCs to ^z^4CL2::STS^His^ functionalized ENCs.

Several strategies for one-step immobilization-purification of enzymes based on the use of antibodies, affinity domains or various ligands were recently reviewed (Barbosa et al., 2015). We anticipated that immobilization of z33-containing enzymes on a virus scaffold would first act as a means to purify the active enzymes from the clarified soluble cell lysate. Unfortunately, a significant amount of contaminating proteins were associated with the assemblies after SDS-PAGE analysis despite the intensive washes (data not shown). This shows a clear need for optimization of the procedure for better capture and binding efficiency of the enzymes. This could be achieved either by adding several tags in tandem or repositioning the tags within the enzymes. Optimally the one-step immobilization-purification approach could provide a cost-effective, fast and reliable way to purify and configure nano-devices like lab on a chip, for industrial applications.

## Conclusion

With increasing understanding of living systems, the scientific community has developed new interest for biologically ordered structures having the potential to become ENCs (Cardinale et al., 2012). It appears that ENCs are easier to position on a support than single enzymes using top-down processes. Because of their highly ordered protein nature, virus structures can be precisely decorated with enzymes and used as ENCs. The preliminary work reported here explores the use of viral ENCs to display enzyme cascades on solid supports by means of an interplay between genetically tagged enzymes, immune-conjugation, two bottom up approaches and DAS-ELISA based top-down adsorption. We confirm here the robustness of the z33-fusion strategy as a method to decorate any virus particles via virus-specific antibodies and its ability to coat these particles with proteins as large as 107 kDa. This study provides us with a proof of concept that the simultaneous purification and positioning of tagged enzymes on ordered solid supports can be achieved. This could be the first step toward a direct way to assemble protein biochips.

## Author Contributions

JB-N, MW, DC, TM, JW, and KM conceived and designed the experiments. JB-N, MW, DC, and JP performed the experiments and JB-N, MW, DC, JP, TM, and KM interpreted the results. JB-N, TM, JW, and KM wrote the paper. All authors discussed the results and commented on the manuscript and have given approval to the final version of the manuscript.

## Conflict of Interest Statement

The authors declare that the research was conducted in the absence of any commercial or financial relationships that could be construed as a potential conflict of interest.
